# *Bifidobacterium* Treated by Electrostatic Spray Drying Relieved Constipation by Changing the Relative Abundance of Bacteria Associated With Gastrointestinal Regulatory Peptides

**DOI:** 10.3389/fcimb.2022.894216

**Published:** 2022-04-27

**Authors:** Tian Jiang, Wenwei Lu, Zhifeng Fang, Hongchao Wang, Jinlin Zhu, Hao Zhang, Jianxin Zhao

**Affiliations:** ^1^ State Key Laboratory of Food Science and Technology, Jiangnan University, Wuxi, China; ^2^ School of Food Science and Technology, Jiangnan University, Wuxi, China; ^3^ National Engineering Research Center for Functional Food, Jiangnan University, Wuxi, China; ^4^ (Yangzhou) Institute of Food Biotechnology, Jiangnan University, Yangzhou, China; ^5^ Wuxi Translational Medicine Research Center and Jiangsu Translational Medicine Research Institute Wuxi Branch, Wuxi, China

**Keywords:** electrostatic spray drying, microencapsulation, *Bifidobacterium*, constipation, intestinal microbiota, aquaporin, gastrointestinal regulatory peptide

## Abstract

In this study, three different microencapsulation methods were used to embed *Bifidobacterium* to explore the alleviating effects of embedding methods on constipated mice. By measuring the defecation-related parameters, it was found that the Bifidobacteria treated by electrostatic spray drying had the best ability to relieved constipation. Furthermore, by detecting constipation-related gastrointestinal regulatory peptides, inflammatory factors, intestinal microbiota, and SCFAs, it was discovered that Bifidobacteria treated by electrostatic spray drying changed the composition of intestinal microbiota, especially the relative abundance of bacteria that were positively correlated with AQP3, but negatively correlated with ET-1 and SS, then increased the level of AQP3 in the intestine, and finally relieved constipation by increasing the fecal water content and small intestinal propulsion rate. In conclusion, the electrostatic spray drying method was superior to the other two methods in maintaining the activity of Bifidobacteria and relieved constipation by changing the relative abundance of bacteria that were correlated with gastrointestinal regulatory peptides and increasing the content of fecal water and small intestinal propulsion rate.

## Introduction

Functional constipation is a common gastrointestinal disease with a global incidence of more than 10% (Johanson and Kralstein, 2007). The application of probiotics to relieve constipation is currently a research hotspot in the treatment of constipation (Horiuchi et al., 2020; Lu et al., 2021; Park et al., 2021; Saviano et al., 2021; Wang et al., 2022). At present, the most effective probiotics for constipation are mainly *Lactobacillu*s and *Bifidobacterium* (Matsumoto et al., 2006; Ishizuka et al., 2012; Wang et al., 2017). For these probiotics to play a role in relieving constipation, the primary condition is live microorganisms and enough (Papagianni and Anastasiadou, 2009; Paéz et al., 2012). However, only a few probiotics can reach the large intestine through the natural barrier of stomach acid. Therefore, improving the stability and survival rate of probiotics through effective technologies is the key to their probiotic functions.

Microcapsules are small capsules that can encapsulate active substances inside micron-sized particles and play a new role in protecting active substances (Rodrigues et al., 2017). Microencapsulation technology has been widely used in related fields such as oil antioxidant, food flavor preservation, pharmaceutical controlled release, food fermentation, and probiotic products (Liu et al., 2016). Microcapsules provide physical protective barriers for microorganisms to isolate them from harsh external environmental conditions and play a role in protecting microorganisms (Chen et al., 2017). The biggest problem in probiotic products is that the extreme environment of the gastrointestinal tract severely reduces the survival rate of probiotics during consumption (Verruck et al., 2017). Microcapsules play an important role in protecting the embedded probiotics from harmful external factors, enhancing the survival level and activity of microorganisms (Fayed et al., 2018). At present, extrusion, emulsification, and spray drying are relatively mature methods (Heidebach et al., 2012). Among them, the extrusion method is simple to operate, but the particle size of obtained microcapsules is larger, and this method cannot produce on a large scale (Broeckx et al., 2016). Emulsification freeze-drying can adjust the size of microcapsules, but this method needs to add an oil phase and is costly (Bosnea et al., 2017). The traditional spray-drying method has a high evaporation temperature, and the active probiotic raw materials are easily inactivated (Fayed et al., 2018). The electrostatic spray-drying method is carried out in the electric field between the positive and negative plates of the DC high-voltage generator, which can avoid the damage to bacteria caused by high temperature (Tapia-Hernandez et al., 2015).

To explore the effect of embedding methods on Bifidobacteria and the ability of microencapsulated Bifidobacteria to relieve constipation, we selected 6 strains of 3 species of Bifidobacteria which have relieving constipation (two strains of *B. animals* subsp. *lactic*, two strains of *B. longum*, and two strains of *B. longum* subsp. *infantis*) and used conventional spray drying, emulsification freeze-drying, and electrostatic spray drying to embed them. Finally, we determined the best embedding method of Bifidobacteria and the mechanism of alleviating constipation in this way by detecting short-chain fatty acids (SCFAs), constipation-related gastrointestinal regulatory peptides, intestinal microbiota, and inflammatory factors.

## Materials and Methods

### *Bifidobacterium* Preparation and Microencapsulation

*Bifidobacterium* strains were from the Culture Collection of Food Microbiology at Jiangnan University (**Table 1**). The *Bifidobacterium* strain was activated in MRS broth with 0.5% L-cysteine hydrochloride (m/v), and bacterial sludges were collected. The obtained bacterial sludge and protective agents (trehalose, linolenic acid, CaHPO_4_, and β-cyclodextrin) were emulsified with a homogenizing speed of 1,000 r/min and a homogenizing time of 10 min. The mass ratio of bacterial sludge and protective agents was 1:2. Then, the obtained solutions were microencapsulated by three different methods. For electrostatic spray drying, the air inlet temperature, the material pump flow, and the electrostatic pressure were set to 80°C, 30 r/min, and 25 kV, respectively (Spraying Systems Co., Naperville, USA). For conventional spray drying, the air inlet temperature was 130°C and the material pump flow was 45 r/min (Armfield Ltd, Ringwood, England). For emulsification freeze-drying, the solutions were pre-frozen at 50°C for 2 h, gradually raised to 18°C, and dried for 24 h. Then, the temperature was raised to 28°C and dried for 4 h (Biocool, Beijing, China). The total cycle was 48 h. The following animal experiments all used the strains treated with these three embedded methods.

**Table 1:** The specific information of the experimental strain.

Treatment	Strains	Abbreviation
Conventional spray drying	*B. animalis* subsp. *lactis* BL03	BL03-1
	*B. animalis* subsp. *lactis* BAL005	BAL005-1
	*B. longum* BLL2	BLL2-1
	*B. longum* BLL11	BLL11-1
	*B. longum* subsp. *infantis* BI20	BI20-1
	*B. longum* subsp. *infantis* BI63	BI63-1
Emulsification freeze-drying	*B. animalis* subsp. *lactis* BL03	BL03-2
	*B. animalis* subsp. *lactis* BAL005	BAL005-2
	*B. longum* BLL2	BLL2-2
	*B. longum* BLL11	BLL11-2
	*B. longum* subsp. *infantis* BI20	BI20-2
	*B. longum* subsp. *infantis* BI63	BI63-2
Electrostatic spray drying	*B. animalis* subsp. *lactis* BL03	BL03-3
	*B. animalis* subsp. *lactis* BAL005	BAL005-3
	*B. longum* BLL2	BLL2-3
	*B. longum* BLL11	BLL11-3
	*B. longum* subsp. *infantis* BI20	BI20-3
	*B. longum* subsp. *infantis* BI63	BI63-3

### Animals and Experimental Design

SPF-grade BALB/c male mice (6-week-old, GemPharmaTech, Nanjing, China) were maintained under controlled conditions (20°C–26°C, 12 h light/12 h dark, and 40%–70% humidity level). After 1 week of adaptation, mice were randomly divided into 21 groups (n = 6) including the control, constipation model, positive control (BB12), and 18 bifidobacterial groups (BL03-1, BAL005-1, BLL2-1, BLL11-1, BI20-1, BI63-1, BL03-2, BAL005-2, BLL2-2, BLL11-2, BI20-2, BI63-2, BL03-3, BAL005-3, BLL2-3, BLL11-3, BI20-3, and BI63-3, **Table 1**). The control group was gavaged with 0.2 ml sterile saline twice a day, and the BB12-positive control group and 0.2 ml microencapsulated Bifidobacteria (2 × 10^8^ CFU/ml) were gavaged per day during the intervention expectation. After that, a 1-week modeling period was entered. The control group was gavaged with 0.2 ml sterile saline twice a day. The BB12-positive control group, the constipation model group, and the microencapsulated Bifidobacteria-treated groups were given 0.2 ml loperamide hydrochloride (10 mg/kg b.w) and then gavaged with 0.2 ml microencapsulated Bifidobacteria (2 × 10^8^ CFU/ml) after 1 h. The control group was gavaged with 0.2 ml sterile saline after 1 h. The mice were killed by removing the cervical vertebra, and blood and gastrointestinal samples were collected and stored at 80°C for use.

### Determination of Fecal Water Content

The determination of the content of fecal water referred to the method of Wang et al. (2022) and was calculated by the following equation:


Fecal water contents = (wet weight-freeze drying weight)/wet weight ×100%

### Measurement of the Time of First Black Stool

Before the end, mice were given activated carbon solution, and the time from the end of gavage to the detection of the first black stool was recorded (Wang et al., 2022).

### Measurement of the Small intestinal Transit Rate

The small intestinal transit rate was determined according to a study of Liu et al. (2017). In general, mice were gavaged with 0.2 ml of activated carbon powder solution for 30 min. Then, mice were killed by removing the cervical vertebra, and the intestinal tubes from the upper end of the pylorus and bottom to the ileocecal bowel were cut. The small intestine was gently pulled into a straight line to measure the length of the pylorus to the forefront of ink, and the small intestinal transit rate was calculated by the following equation:


$$Small\;intestinal\;transit\;rate=\frac{Distance\;travelled\;by\;the\;activated\;carbon\;meal}{Total\;length\;of\;small\;intestinal\;segments}\times 100\%$$

### Regulatory Peptides of the Gut Measurement

Regulatory peptides of the gut levels (endothelin-1, ET-1; substance P, SP; gastrin, GAS; somatostatin, SS; motilin, MT; vasoactive intestinal peptide, VIP) in serum were measured using an enzyme-linked immunosorbent assay (ELISA) kit (Wenle Biotechnology Co., Ltd., Shanghai, China) following the instructions. The contents of aquaporin 3 (AQP 3), occludin, interleukin-1β (IL-1β), and tumor necrosis factor (TNF-α) were measured by using the method described in a previous work (Wang et al., 2022). Colon samples were treated with saline and centrifuged. After centrifugation, the supernatant was collected and measured with ELISA kits.

### Quantified SCFAs in Feces

SCFAs were quantified using gas chromatography–mass spectrometry (GCMS-QP2010 Ultra, Shimadzu Corp., Kyoto, Japan) based on the external standard method and referring to a previous study of Mao et al. (2016). Briefly, colonic contents were treated using the saturated NaCl solution (Sinopharm, Shanghai, China), acidified with 10% sulfuric acid (Sinopharm), and extracted with ether (Sinopharm). Supernatant was collected for GC-MS analysis. The standards of SCFAs (Sigma-Aldrich, St. Louis, MO, USA) were mixed and determined with the same conditions to build a standard curve.

### 16S rDNA Sequencing and Bioinformatics Analysis

To compare the effects of different microencapsulation methods on intestinal microbiota of constipated mice, the 16S rRNA sequencing was carried out. Briefly, the meta-genomic DNA from mouse feces were extracted using the FastDNA Spin Kit for Feces (MP Biomedicals, Irvine, CA, USA). The V3–V4 regions were amplified using the 341F/806R primers (Wang et al., 2022). After purification and quantification of PCR products, the production was sequenced using the Illumina MiSeq platform. The QIIME2 pipeline with DADA2 was used for demultiplexing and quality filtering of the original data (Callahan et al., 2016). The α diversity was evaluated by the Chao1, Observed species, Shannon, and Simpson. The Spearman’s rank correlation coefficient was used to assess the correlation between gut microbiota and pathological indicators (Wang et al., 2022). Sequence data were deposited in the Sequence Read Archive database as BioProject ID: PRJNA816303.

## Results

### Three Different Embedding Methods Had Different Effects on Defecation-Related Parameters

**Figure 1:**
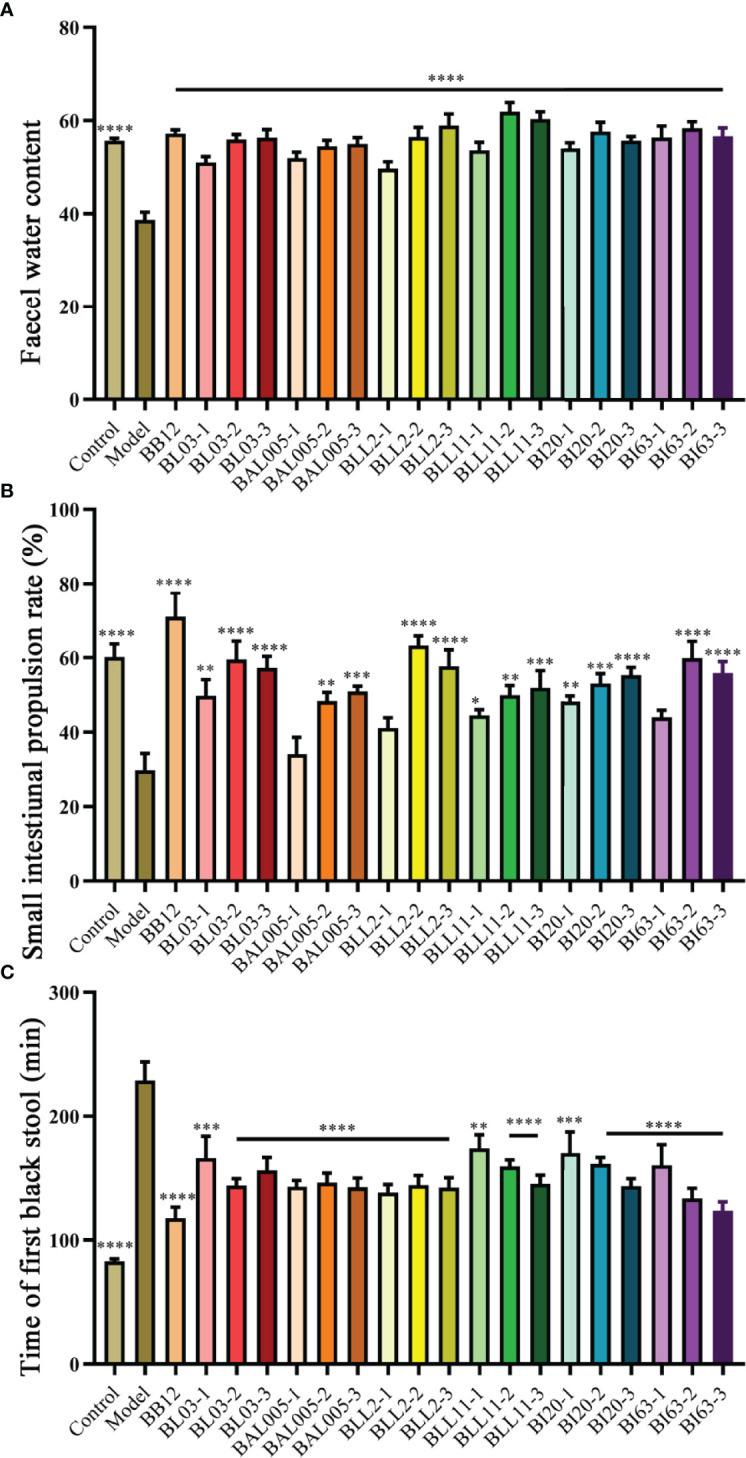
Defecation-related parameters. Fecal water content **(A)**. Small intestinal propulsion rate **(B)**. Time of first black stool **(C)**. There is a significant difference between the bacteria intervention group and the model group, where * means P < 0.05, **P < 0.01, ***P < 0.005, ****P < 0.001.

The fecal water content (**Figure 1A**), the small intestinal propulsion rate (**Figure 1B**), and the time of first black stool (**Figure 1C**) were analyzed to evaluate whether microencapsulated Bifidobacteria could relieve constipation in mice, and the different effects of embedding methods on defecation-related parameters. The results showed that the fecal water content of the model group was decreased, and the small intestine propulsion rate and the time of fist black stool were significantly increased. Six strains of *Bifidobacterium* treated by three different embedding methods significantly alleviated all defecation-related parameters, while the small intestinal transit rate was not significantly different in the BAL005-1, BLL2-1, and BI63-1 groups, and the model group. Among the three different embedding methods, electrostatic spray drying showed the best ability to relieve defecation-related parameters. This may be due to the higher encapsulation rate of Bifidobacteria and better resistance to adverse environments by electrostatic spray drying (Jiang et al., 2021).

### Embedding Methods Affected the Expression of AQP3 in Serum

**Figure 2:**
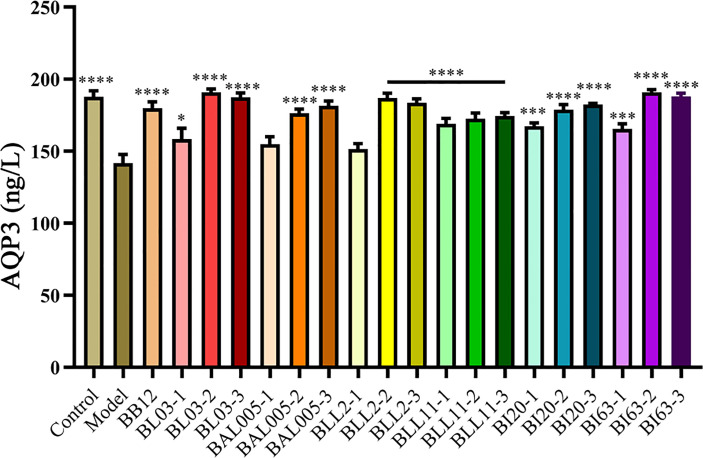
The expression of AQP3 in serum. There is a significant difference between the bacterial intervention group and the model group, where * means P < 0.05, ***P < 0.005, ****P < 0.001.

Aquaporins (AQPs) are a kind of cell membrane proteins that control the transmission of water and some small molecular substances, so that water molecules can be selectively absorbed and secreted to maintain the water balance of the intestine. Therefore, there is an important correlation between the decrease in fecal water content and the abnormal expression of AQPs in patients with constipation (Zhou et al., 2016). As shown in **Figure 2**, the expression of AQP3 in serum was strikingly decreased (P < 0.001). In the Bifidobacteria-intervention group, six strains of *Bifidobacterium* treated by three different embedding methods significantly increased the expression of AQP3 in serum (P < 0.05), but only the expression of AQP3 in the BAL005-1 and BLL2-1 groups was not significantly different from the model group.

### Embedding Methods Improved Constipation-Related Gastrointestinal Regulatory Peptides

**Figure 3:**
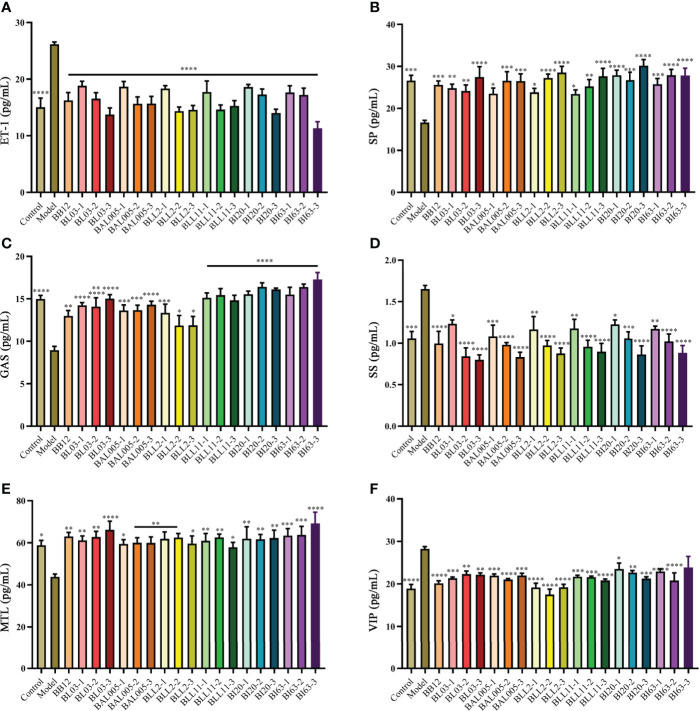
The levels of gastrointestinal regulatory peptides in serum of mice. ET-1 **(A)**. SP **(B)**. GAS **(C)**. SS **(D)**. MTL **(E)**. VIP **(F)**. There is a significant difference between the bacterial intervention group and the model group, where * means P < 0.05, **P < 0.01, ***P < 0.005, ****P < 0.001.

As shown in **Figure 3**, some gastrointestinal regulatory peptides related to constipation were determined, such as ET-1 (**Figure 3A**), SP (**Figure 3B**), GAS (**Figure 3C**), SS (**Figure 3D**), MTL (**Figure 3E**), and VIP (**Figure 3F**). These peptides can significantly regulate gastrointestinal motility. Compared to the model group, the levels of excitatory gastrointestinal regulatory peptides GAS (**Figure 3C**), MTL (**Figure 3E**), and SP (**Figure 3B**) in the serum of constipated mice were significantly increased in varying degrees. On the contrary, the levels of inhibitory gastrointestinal regulatory peptides ET-1 (**Figure 3A**), SS (**Figure 3D**), VIP (**Figure 3F**) in the serum were significantly decreased in different drying treatment groups, which showed the same trend as the positive control group (*B. animalis* BB12). These results indicated that the abovementioned probiotics treated with different embedding methods effectively improved the relevant indicators caused by constipation.

### The Strains Treated With the Electrostatic Spray Drying Method Upregulated the Content of SCFAs

**Figure 4:**
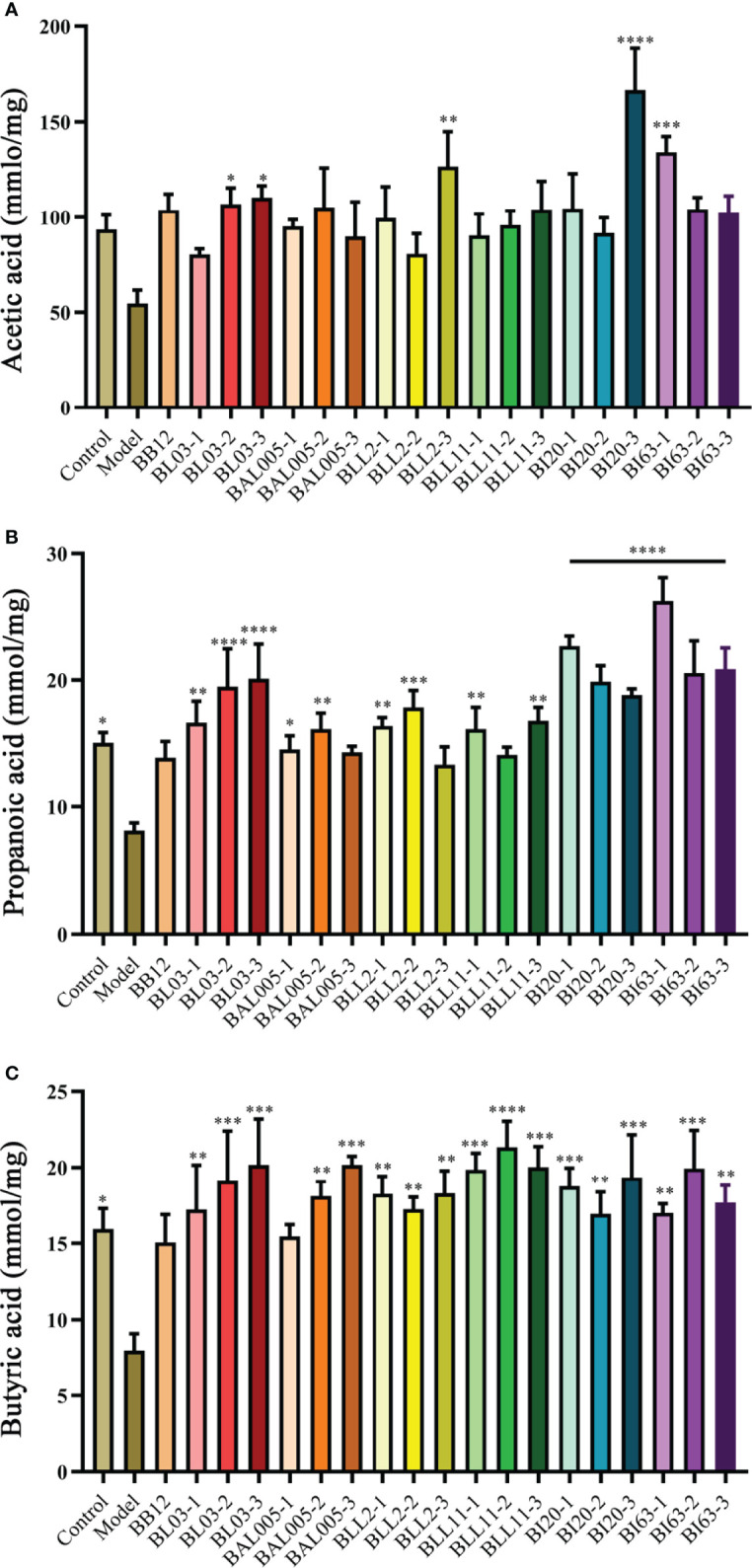
The levels of SCFAs in mice feces. Acetic acid **(A)**. Propionic acid **(B)**. Butyric acid **(C)**. There is a significant difference between the bacterial intervention group and the model group, where * means P < 0.05, **P < 0.01, ***P < 0.005, ****P < 0.001.

Compared with the control group, acetic acid (**Figure 4A**), propionic acid (**Figure 4B**), and butyric acid (**Figure 4C**) were reduced in the model group, and propionic acid and butyric acid were significantly decreased (P < 0.05). The content of total fecal SCFAs of the positive control group had an upward trend. No matter which embedded methods used, the contents of propionic acid and butyric acid in mouse feces were obviously increased. However, different embedded methods had different effects on acetic acid. After treating by conventional spray drying, the content of acetic caid of the *B. animalis subsp. lactis* BL03 group was reduced, while emulsification freeze drying and electrostatic spray drying increased the content of acetic acid (P < 0.05). Conventional spray drying significantly improved the acetic acid content of the *B. infantis* BI63 group (P < 0.05). After electrostatic spray drying, the level of acetic acid of the *B. infantis* BI20 group was extremely significantly increased (P < 0.001), and that of *B. animalis* subsp. *lactis* BL03 and *B. longum* BLL2 groups was obviously increased (P < 0.05). Based on these results, the strains treated by electrospray drying significantly increased the content of SCFAs in mouse feces.

### Embedding Methods Had No Effect on the Intestinal Barrier and the Levels of Inflammatory Factors

**Figure 5:**
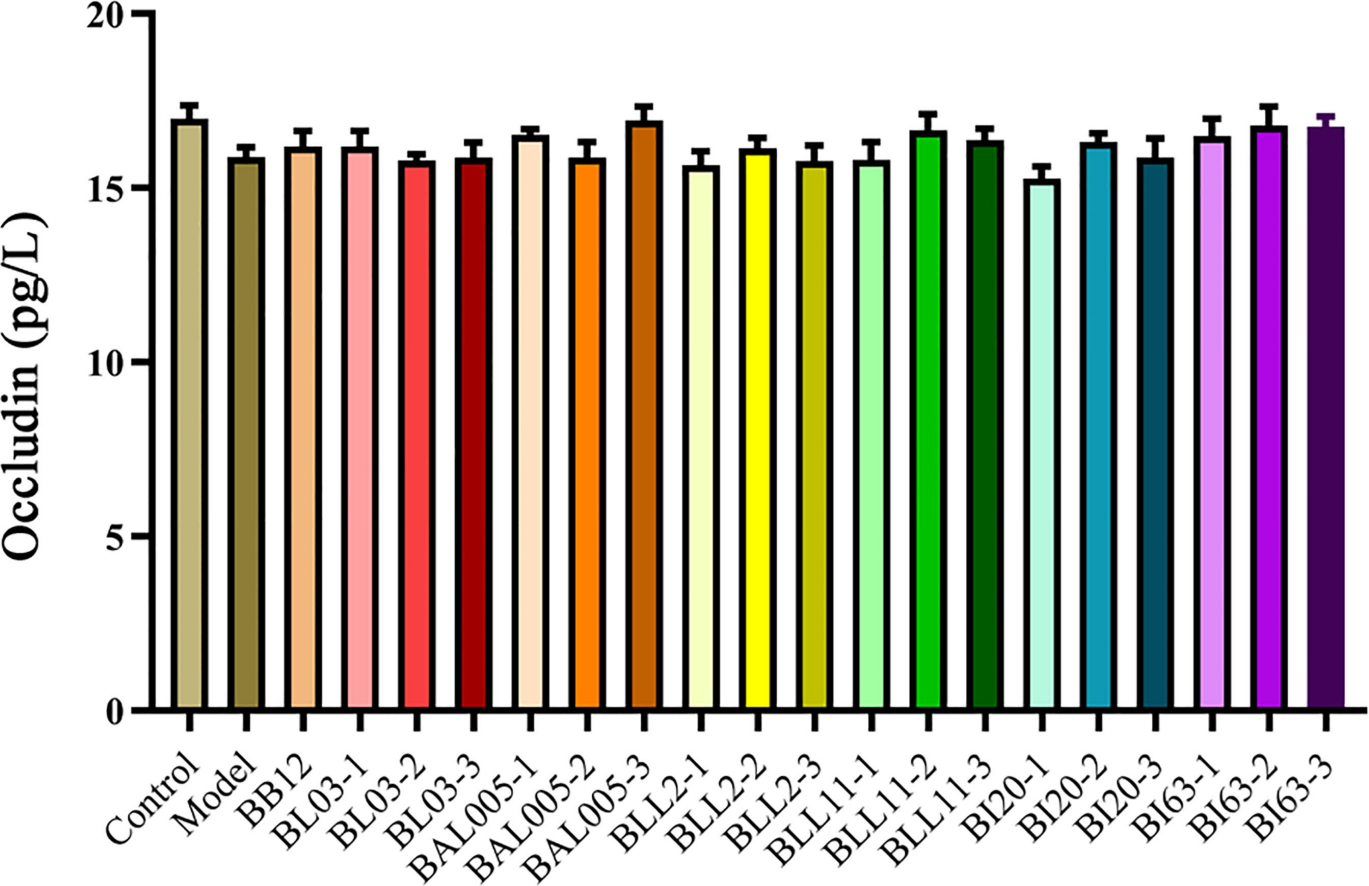
The occludin levels in serum of mice treated with different embedding methods.

Occludin can maintain intestinal integrity, and the lacking of occludin may aggravate the damage of intestinal barrier function (Wang et al., 2022). Based on the analysis shown in **Figure 5**, the expression of occludin in the constipation model group showed a downward trend compared to the control group. The strains treated by different embedding methods had almost no effect on the level of occludin in serum of constipated mice. It could be seen that constipation had almost no effect on the intestinal barrier of mice, and the strains treated by different embedding methods also had almost no effect on the intestinal barrier.

**Figure 6:**
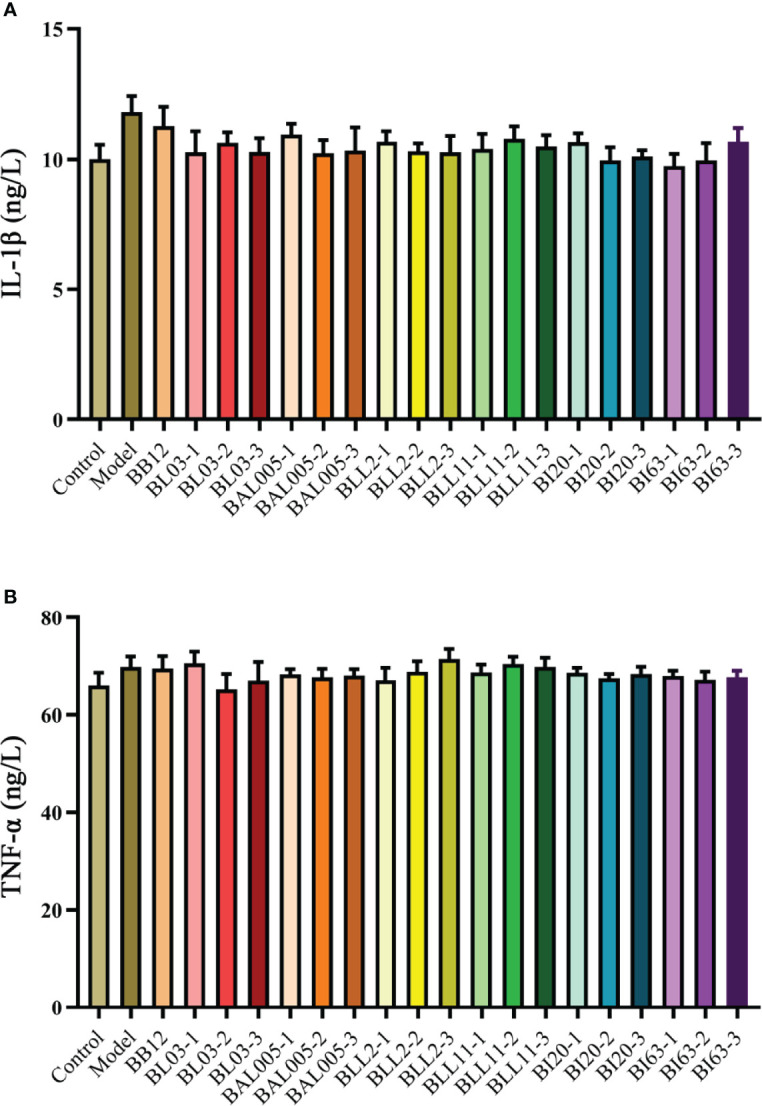
The levels of IL-1β **(A)** and TNF-α **(B)** in serum of mice treated with different embedding methods.

In addition to occludin, the effects of the embedding method on intestinal inflammatory factors (IL-1β and TNF-α) were also considered. The measurement results of these two inflammatory factors are shown in **Figure 6**. There was no significant difference in the levels of IL-1β (**Figure 6A**) and TNF-α (**Figure 6B**) among the control, model, positive control, and different bacterial treatment groups. This showed that the strains treated with different embedding methods also had little effect on the level of inflammatory factors.

### The Bifidobacteria Treated by Electrostatic Spray Drying Relieved Constipation by Changing the Composition of Intestinal Microbiota

**Figure 7:**
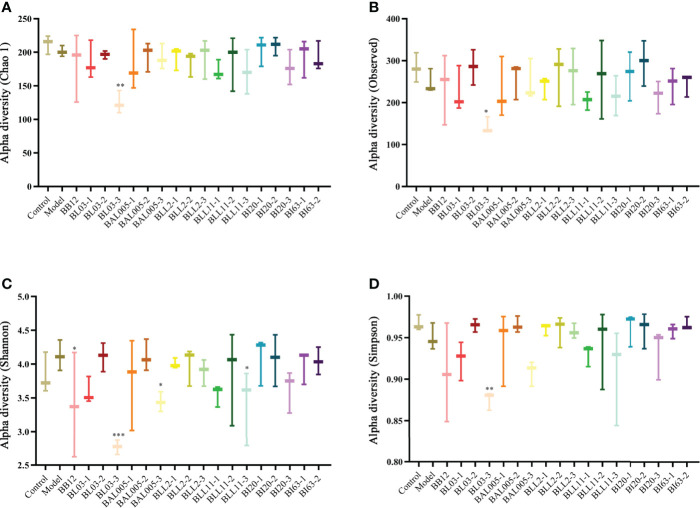
The alpha diversity of intestinal microbiota in mice treated with different embedding methods. Chao 1 index **(A)**. Observed species index **(B)**. Shannon index **(C)**. Simpson index **(D)**, where * means P < 0.05, **P < 0.01, ***P < 0.005.

Microencapsulation provides good protection for *Bifidobacterium* and separates it from adverse environments. According to our previous study, the embedding rate of *Bifidobacterium* after microencapsulating can reach 93.31 ± 3.16%, and the survival rate can reach more than 50% after simulating the digestive tract (Jiang et al., 2021). We gavaged constipated mice with microencapsulated *Bifidobacterium* and determined the effects on gut microbiota. The Chao1 (**Figure 7A**), Observed species (**Figure 7B**), Shannon (**Figure 7C**), and Simpson (**Figure 7D**) indexes were used to analyze the α-diversity. Compared to the control group, the Chao1, Observed species, and Simpson indexes showed a downward trend in the model group. The alpha diversity of six strains of *Bifidobacterium* treated by three different embedding methods showed varying degrees of variation relative to the model group. Except for the BAL005-1 group, the other groups had no significant difference with the model group. There was no significant difference in beta diversity between Bifidobacteria-intervention groups and the model group (data were not shown).

**Figure 8:**
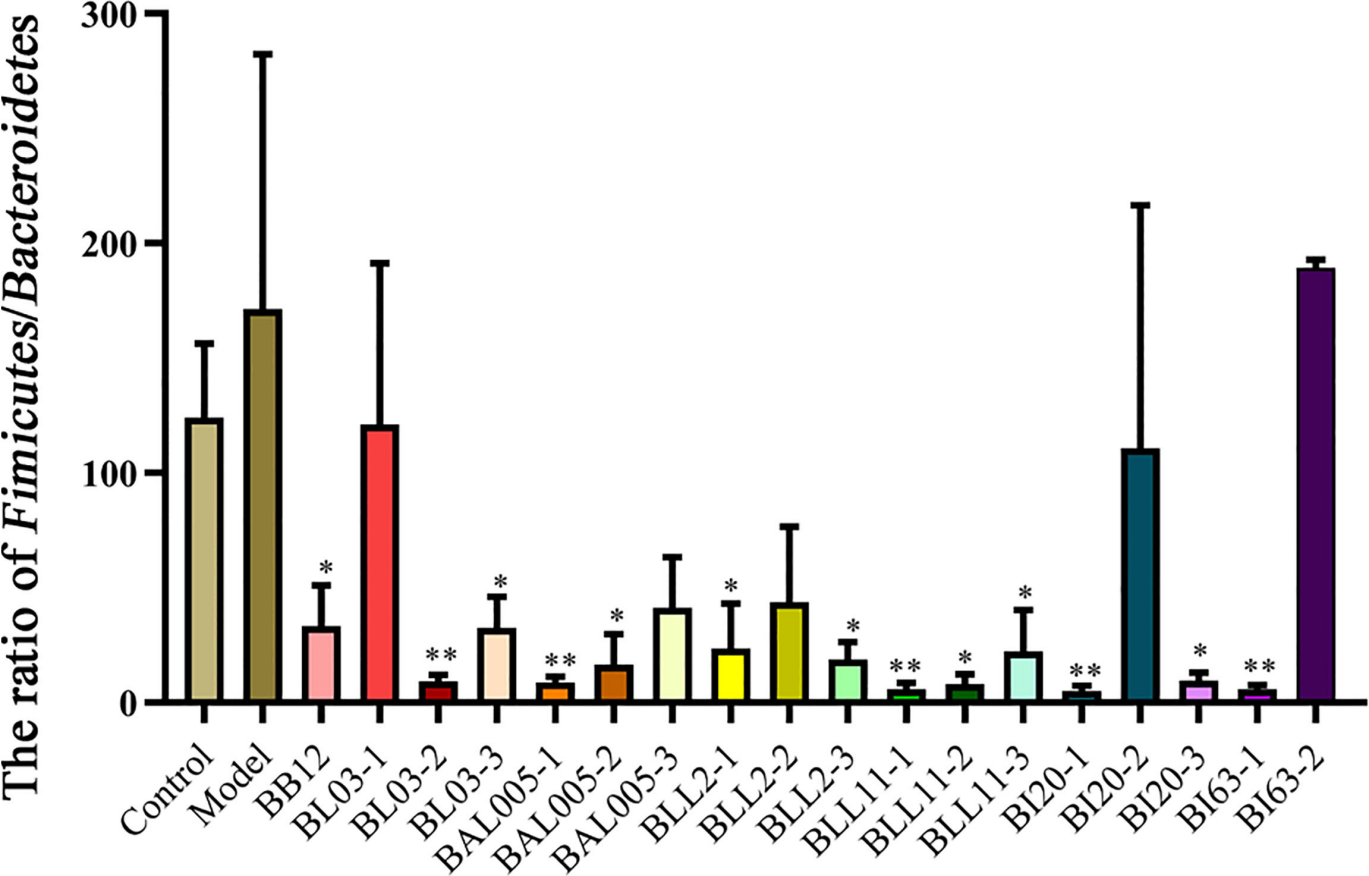
The ratio of *Firmicutes*/*Bacteroidetes* in mice treated with different embedding methods. There is a significant difference between the bacterial intervention group and the model group, where * means P < 0.05, **P < 0.01.

At the phylum level, the relative abundance of *Bacteroides* in the constipated model group was lower, while the relative abundance of *Firmicutes* was higher, and the ratio of *Firmicutes*/*Bacteroidetes* was higher, whereas the ratio of *Firmicutes*/*Bacteroidetes* of different embedding method-treated Bifidobacteria-intervention groups was lower (**Figure 8**). This indicated that Bifidobacteria regulated the intestinal microbiota.

**Figure 9:**
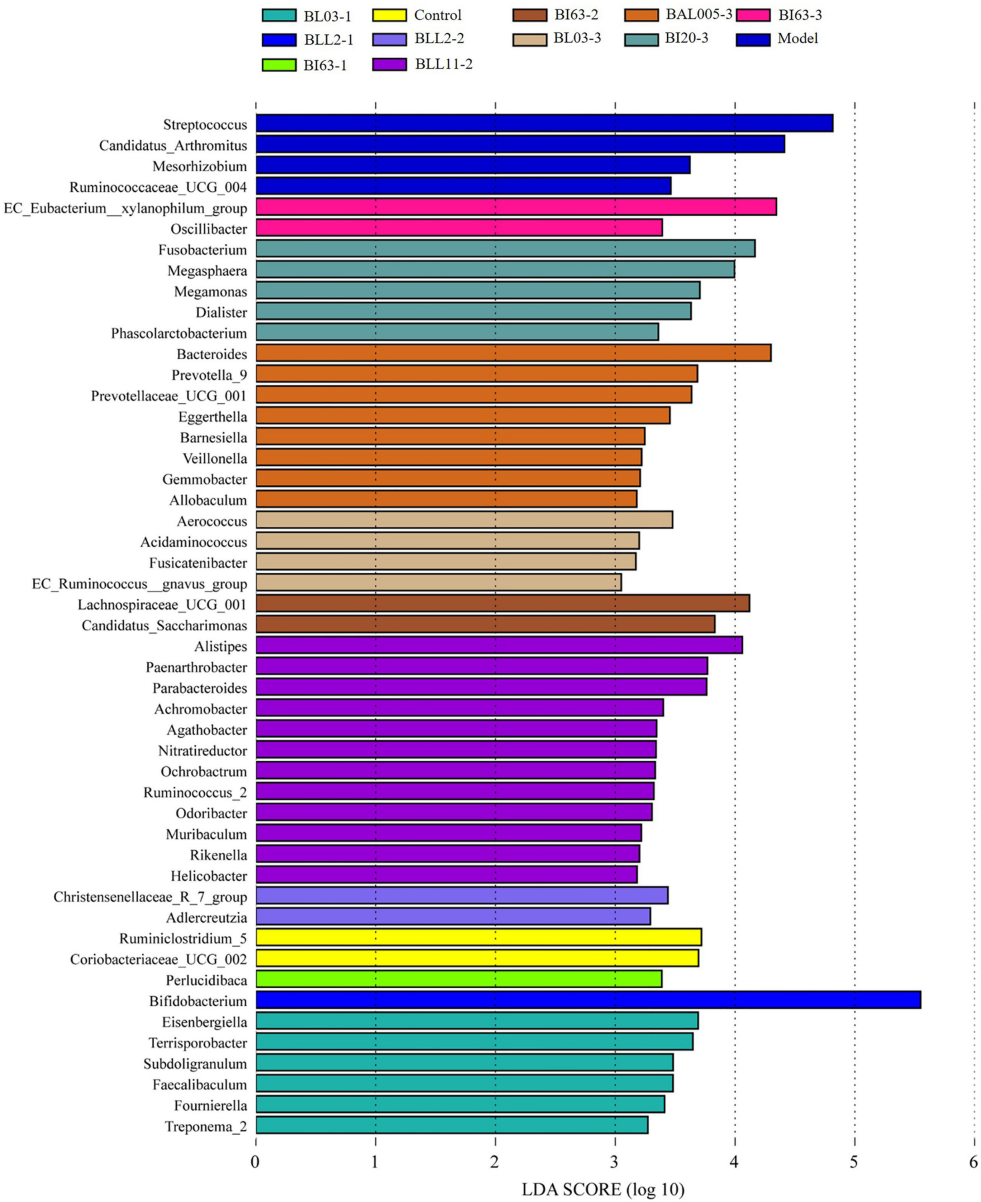
The LEfSe analysis of gut microbiota in mice treated with different embedding methods.

Further, we carried out LDA Effect Size (LEfSe) analysis on the fecal microbiota data of mice and obtained biomarkers (LDA value > 2) with statistical differences in the mouse gut. The results are shown in **Figure 9**. The characteristic genera of the intestinal microbiota in the model group were *Streptococcus*, *Ruminococcaceae* UGG004, and *Candidatus_Arthromitus*. The characteristic genera of the control group were *Ruminiclostridium* 5 and *Coriobacteriaceae* UCG002. Bifidobacteria treated with different embedding methods had different effects on the intestinal microbiota of mice after intervention in constipated mice. Among them, the strains treated by the electrostatic spray-drying method had the greatest influence on the composition of the intestinal microbiota of the constipated mice, followed by the emulsification freeze-drying method, and finally the conventional spray-drying method. The biomarkers with significant differences in the electrostatic spray-drying groups were much larger than those in the emulsification freeze-drying and conventional spray-drying groups. Among them, *Lachnospiraceae* UCG001, *Aerococcus*, *Bacteroides*, *Fusobacterium*, and *EC_Eubacterium_xylanophilum*_group played an important role in the electrostatic spray-drying group.

**Figure 10:**
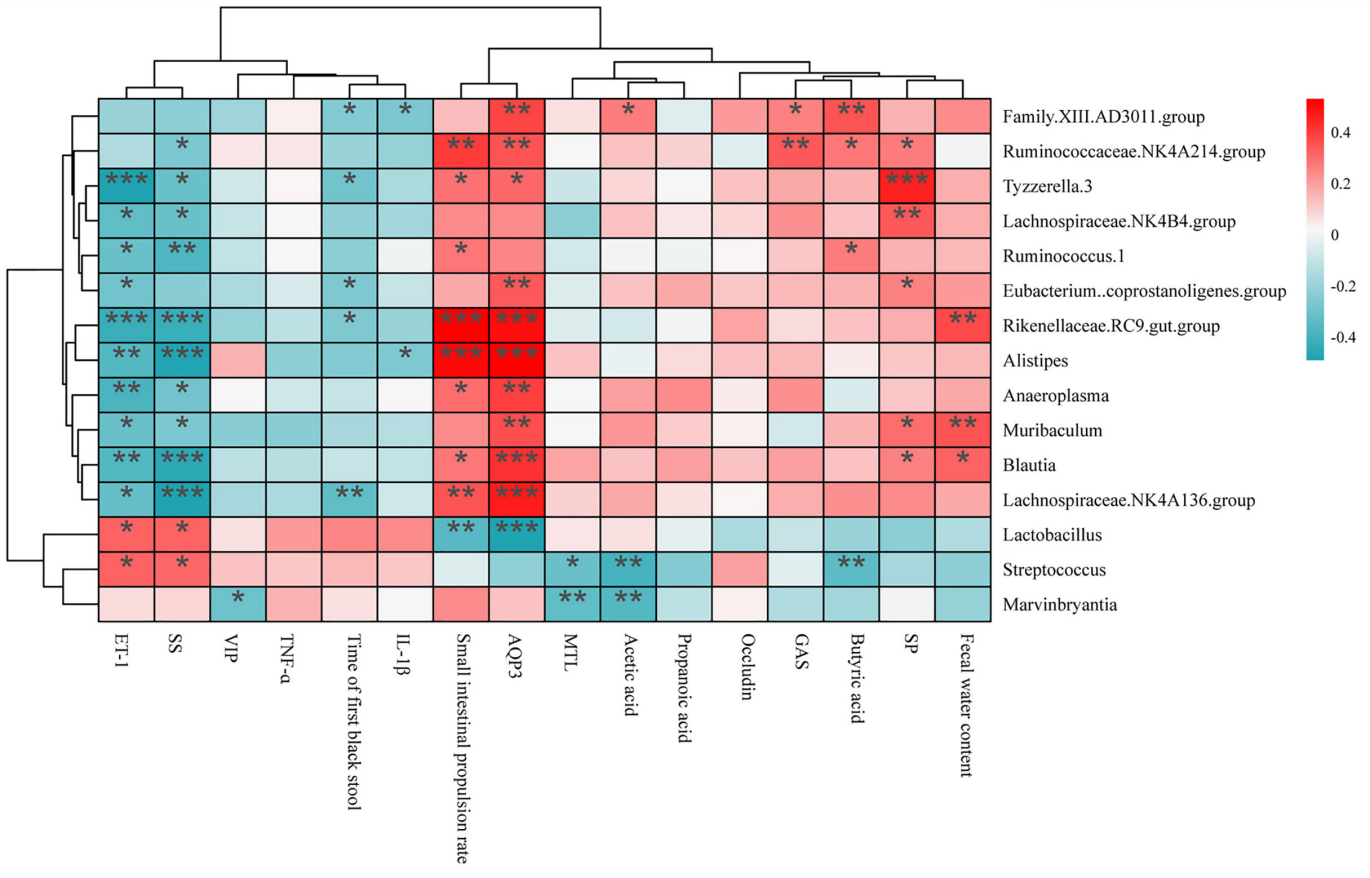
Spearman’s correlation analysis between changed bacterial genera and constipation-related indicators. The colors ranged from blue (negative correlation) to red (positive correlation), and significant correlations were marked by *P < 0.05, **P < 0.01, ***P < 0.001.

The correlation analysis is shown in **Figure 10**. There were 12 bacterial genera (*Ruminococcaceae* NK4A214 group, *Family.XIII.*AD3011 group, *Rikenellaceae. *RC9*.gut* group, *Alistipes*, *Anaeroplasma*, *Ruminococcus.*1, *Eubacterium.coprostanoligenes* group, *Tyzzerella.3*, *Lachnospiraceae.*NK4B4 group, *Muribaculum*, *Lachnospiraceae* NK4A136 group, and *Blautia*) positively related to the water content of feces, small intestine propulsion rate, acetic acid, propionic acid, and AQP3. However, these bacterial genera were negatively related to the time of the first black stool, ET-1 and SS. Among them, 3 genera (*Rikenellaceae.RC9.gut.group*, *Muribaculum*, and *Blautia*) were significantly positively related to the fecal water content, 8 genera (*Ruminococcaceae* NK4A214 group, *Alistipes*, *Rikenellaceae.*RC9 gut group, *Anaeroplasma*, *Ruminococcus.*1, *Tyzzerella.*3, *Blautia*, and *Lachnospiraceae.*NK4A136 group) were significantly positively correlated with the intestinal propulsion rate, and 5 genera (*Family.XIII.*AD3011 group, *Rikenellaceae.*RC9*.gut* group, *Eubacterium.coprostanoligenes* group, *Tyzzerella.*3, and *Lachnospiraceae.*NK4A136 group) were significantly negatively correlated with time to the first black stool defecation.

## Discussion

Constipation is a functional gastrointestinal disease, and its occurrence is related to age, living habits, mental state, and psychological factors (Liu et al., 2020; Demler and Krieger, 2021). Severe constipation can cause gastrointestinal nerve dysfunction and even induce cancer and cardiovascular and cerebrovascular diseases (Nishitani-Yokoyama et al., 2021; Huang et al., 2022). A large number of studies have shown that probiotics can shorten gastric emptying time and intestinal transit time, enhance the peristaltic transmission function of the gastrointestinal tract, and help relieve constipation (Zhang et al., 2021). We selected Bifidobacteria treated with different embedding methods to determine whether the embedding method affected the strains’ effectiveness in relieving constipation. The results showed that the Bifidobacteria treated by the conventional spray-drying method could not relieve constipation, while the Bifidobacteria treated by the electrostatic spray-drying method and emulsification freeze-drying method could relieve constipation. Different microencapsulation methods of Bifidobacteria changed the fecal water content, small intestinal transit time, and SCFA content in feces and affected constipation-related gastrointestinal regulatory peptides, inflammatory factors, and the composition of intestinal microbiota.

By detecting the expression of AQP3, it was found that microencapsulation can increase the level of AQP3 in serum. Among them, emulsified freeze-drying and electrostatic spray microencapsulation of Bifidobacteria showed better AQP3-upgrading ability. AQPs is a cell membrane channel protein and is closely associated with excessive water absorption and mucus secretion reduction in colon. Therefore, AQP regulation is an important way to treat diarrhea and constipation (Zhou et al., 2016). AQP3 is the most closely related to constipation in the current research (Matsuzaki et al., 2004) and plays a key role in the modulation of colonic fluid metabolism. AQP3 is abundantly expressed in epithelial cells of the digestive tract and is mainly involved in the transport and metabolism of fluid and electrolytes in the digestive tract. Previous studies had found that regulating the VIP-cAMP-PKA-AQP3 signaling pathway can relieve constipation (Zhou et al., 2016). It is speculated that the Bifidobacteria treated by electrostatic spray drying may relieve constipation by regulating the above signaling pathway, which is the focus of our next research.

SCFAs are produced by anaerobic colonic bacteria that ferment oligosaccharides that are not absorbed by the small intestine. It has a two-way regulation of inhibition and excitement of intestinal motility (Cherbut et al., 1997). As early as the last century, scientists had noticed that SCFAs can affect intestinal motility. Even though SCFAs cannot directly touch the small intestine, they might participate in the “ileocolonic brake,” which means that nutrients reach the junction of the ileum and cecum to inhibit gastric emptying (Dass et al., 2007), and studies have shown that SCFAs can increase the frequency of peristalsis and contraction of the terminal ileum of guinea pigs, thereby affecting intestinal motility (Li et al., 2015). In the research, the levels of SCFAs decreased in constipated mice. Acetate levels increased after electrostatic spray-drying treatment. Chen et al. (2019) obtained similar results using *Lactobacillus plantarum* NCU116 to relieve constipation and proved that the content of acetic acid in mouse feces had a certain dose–effect relationship with *Lactobacillus plantarum* NCU116.

Microencapsulated Bifidobacteria significantly increased the level of excitatory gastrointestinal regulatory peptides in the serum of constipated mice. At the same time, the level of inhibitory gastrointestinal regulatory peptides was reduced. However, there was no significant effect on the level of occludin and inflammatory factors (IL-1β and TNF-α) in constipated mice after gavage with microencapsulated Bifidobacteria. Therefore, microencapsulation methods have no significant difference in altering the intestinal barrier and alleviating inflammation in constipated mice. The destruction of the intestinal barrier may cause more serious intestinal bacterial imbalance, which will lead to more gastrointestinal diseases (Wang et al., 2022).

Microencapsulation of Bifidobacteria reduced the ratio of the ratio of *Firmicutes*/*Bacteroides* related to fecal hardness, and the electrostatic spray-drying method showed the best effect. This suggests that the mechanism of Bifidobacteria alleviating constipation may be related to the ratio of *Firmicutes*/*Bacteroides*. We found an interesting result when the gut microbiota was co-analyzed with constipation-related gastrointestinal regulatory peptides, AQP3, fecal water content, and small intestinal transit time. Almost all the bacteria that were significantly positively correlated with the small intestinal propulsion rate were positively correlated with AQP3 but negatively correlated with ET-1 and SS. Combined with the influence of Bifidobacteria treated by three microencapsulated methods on constipation, Bifidobacteria treated by electrostatic spray drying changed the composition of intestinal microflora, and especially positively correlated with AQP3, but negatively correlated with ET-1 and SS. It increases the level of AQP3 in the intestine, and finally alleviates constipation by increasing the content of fecal water and the small intestinal propulsion rate.

## Data Availability Statement

The datasets presented in this study can be found in online repositories. The names of the repository/repositories and accession number(s) can be found as follows: https://www.ncbi.nlm.nih.gov/, BioProject ID: PRJNA816303.

## Ethics Statement

The animal experiments were approved by the Ethics Committee of Experimental Animals of Jiangnan University (JN. No. 20201030b1681220[294]).

## Author Contributions

TJ, WL, and HZ conceived and designed the experiments. TJ performed the experiments and drafted the manuscript. TJ, HZ, JlZ, and JxZ analyzed the data. JxZ, HW, and ZF contributed the reagents/materials/analysis tools. All authors contributed to the article and approved the submitted version.

## Funding

This work was supported by the National Natural Science Foundation of China (No. 31820103010) and Collaborative Innovation Center of Food Safety and Quality Control in Jiangsu Province.

## Conflict of Interest

The authors declare that the research was conducted in the absence of any commercial or financial relationships that could be construed as a potential conflict of interest.

## Publisher’s Note

All claims expressed in this article are solely those of the authors and do not necessarily represent those of their affiliated organizations, or those of the publisher, the editors and the reviewers. Any product that may be evaluated in this article, or claim that may be made by its manufacturer, is not guaranteed or endorsed by the publisher.
